# Intracellular Host Restriction of Hepatitis B Virus Replication

**DOI:** 10.3390/v16050764

**Published:** 2024-05-11

**Authors:** Prakriti Sinha, Chloe L. Thio, Ashwin Balagopal

**Affiliations:** Department of Medicine, Division of Infectious Diseases, Johns Hopkins University School of Medicine, Baltimore, MD 21205, USA; psinha8@jhmi.edu (P.S.); cthio@jhmi.edu (C.L.T.)

**Keywords:** hepatitis B virus, host restriction factors, viral transcription, cccDNA transcriptional silencing, Smc5/6

## Abstract

The hepatitis B virus (HBV) infects hepatocytes and hijacks host cellular mechanisms for its replication. Host proteins can be frontline effectors of the cell’s defense and restrict viral replication by impeding multiple steps during its intracellular lifecycle. This review summarizes many of the well-described restriction factors, their mechanisms of restriction, and counteractive measures of HBV, with a special focus on viral transcription. We discuss some of the limitations and knowledge gaps about the restriction factors, highlighting how these factors may be harnessed to facilitate therapeutic strategies against HBV.

## 1. Introduction

Chronic hepatitis B (CHB) is a leading cause of liver cirrhosis and hepatocellular carcinoma worldwide. Despite an effective vaccine, CHB leads to >800,000 deaths annually from among approximately 300 million infected individuals. HBV replication can be controlled with treatment but rebounds upon treatment discontinuation; thus, an HBV cure is needed. An HBV cure is challenging because of the lifelong persistence of the viral genomic template—covalently closed circular DNA (cccDNA)—that resides in infected hepatocytes. Although the complete eradication of every hepatocyte with cccDNA is difficult, it may be possible to silence cccDNA transcription, resulting in a functional cure. In recent years, several host proteins have been described that restrict HBV replication. Understanding the host restriction of HBV may illuminate pathways and mechanisms that can be exploited to permanently silence cccDNA transcription and induce an HBV cure.

**Figure 1:**
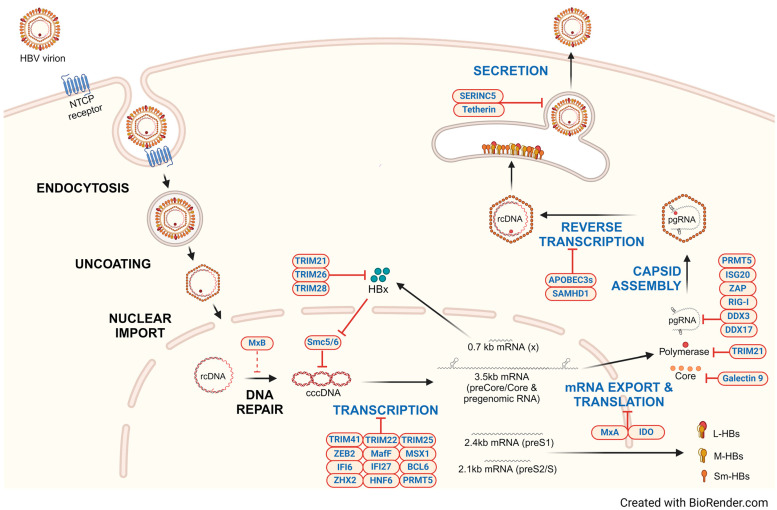
Host restriction factors inhibit distinct steps in the HBV life cycle. Hepatitis B virions bind the NTCP receptor and are endocytosed. After uncoating, rcDNA is transported into the nucleus. Once in the nucleus, rcDNA is repaired by host enzymes to form cccDNA. cccDNA is transcribed by host polymerases into the suite of viral RNAs that are translated into all viral proteins that are required for replication. In addition, pgRNA is transcribed from cccDNA: pgRNA is the template that is encapsidated in new virions along with HBV Pol. Reverse transcription of pgRNA results in rcDNA. Surface antigens embedded in the host TGN form the envelope of the nascent virion. The infectious virion is then secreted from the hepatocyte. Host factors that impede distinct steps in the viral lifecycle are shown in blue. The majority of host restriction factors that have been identified either diminish cccDNA-derived transcription or target already transcribed viral mRNAs, leading to their degradation.

Generally, upon viral infection, host cells mount intracellular defenses to resist or attenuate infection. Intracellular innate immune defenses include sensors that detect foreign viral molecular patterns, such as the toll-like receptors (TLRs), RIG-like receptors (RLRs), NOD-like receptors (NLRs), and the cGAS/STING pathway [1]. However, HBV is largely believed to evade innate immune detection during natural infection [2,3]. Nonetheless, several groups have reported that HBV can be sensed by intracellular mediators such as TLR2 [4], RIG-I [5], or IFI16 [6]. Recognition by these host pathogen recognition receptors (PRRs) can then recruit diverse messenger proteins and activate antiviral mechanisms that restrict virus replication. These effector proteins are antiviral factors, commonly termed host restriction factors, and can target and dampen different stages of the viral lifecycle. Viruses, including HBV, are known to evade many of the restriction factors.Herein, we first review the HBV life cycle (Figure 1) and then discuss the major host factors that restrict different stages of the life cycle and HBV counter-actions (Table 1), with a special emphasis on factors that target viral transcription. We also discuss novel therapeutic strategies that engage several of these factors to control HBV.

## 2. Overview of the HBV Life Cycle

HBV virions infect hepatocytes, entering via high-affinity binding between viral surface proteins on the infectious virion and the sodium taurocholate cotransporting peptide (NTCP) on the hepatocyte surface (Figure 1). This interaction triggers the endocytosis of the virions, after which the capsid is imported into the nuclear pore complex. After uncoating in the nuclear pore, the rcDNA genome is released in the nucleus where it exploits host enzymes to convert the relaxed circular DNA (rcDNA) genome to form cccDNA. cccDNA is assembled with histones and exists as a stable episomal template from which viral RNAs are transcribed. Distinct host transcription factors and enzymes associate with the four promoters and two enhancers (Enh I and II) on the 3.2 kb cccDNA to generate canonical transcripts of four different lengths (3.5 kb, 2.4 kb, 2.1 kb, and 0.7 kb), driven by host RNA Pol II. These viral transcripts are then transported to the cytoplasm and translated into seven HBV proteins: hepatitis B e antigen (HBeAg), hepatitis B core protein (HBc), polymerase (Pol), large hepatitis B surface protein (L-HBs), middle hepatitis B surface protein (M-HBs), small hepatitis B surface protein (Sm-HBs), and the hepatitis B x protein (HBx). Notably, HBx has major roles in maintaining and enhancing cccDNA transcriptional activity (reviewed below). After translation, core proteins oligomerize around HBV Pol and the 3.5 kb pre-genomic RNA (pgRNA) to form a capsid. Reverse transcription by Pol of pgRNA to rcDNA occurs within the capsid by first synthesizing a complementary negative single strand while simultaneously degrading the pgRNA template and then by synthesizing the second positive strand DNA. The second strand synthesis terminates before completion, rendering a partially double-stranded rcDNA in the infectious virion that is then released from the cell, initiating a new cycle of infection of hepatocytes. However, some of the rcDNA-containing capsids may shuttle back to the nucleus to sustain a cccDNA pool in the cell [7]. Host restriction factors inhibit many of these steps in the HBV life cycle. We begin with host restriction of viral transcription, which may be a key step in silencing cccDNA.

## 3. Transcription

### 3.1. Smc5/6

The structural maintenance of chromosome (SMC) 5/6 complex belongs to a conserved family of proteins responsible for the organization and maintenance of genetic material in cells. Multiple proteins comprise this complex: Smc5, Smc6, non-SMC elements (NSE) 1 to 4, and Smc5/6 localization factors (SLF) 1 and 2. Together, these proteins form a ring-like structure, bind to DNA, and use energy from ATP hydrolysis to organize the DNA into manageable folds through a process called loop extrusion, aiding in genomic DNA repair, recombination, and replication [8,9,10]. Smc5/6 has potent antiviral activity against several viruses, including HBV, mainly through the inactivation of their episomal forms [11].

The Smc5/6 complex has been reported to repress cccDNA transcription in multiple in vitro studies in cell lines and primary human hepatocytes [12,13,14]. The exact mechanism of the transcriptional repression of cccDNA is still not fully elucidated, although several studies have uncovered parts of the HBV–Smc5/6 interaction preceding its silencing. In vitro, Smc5/6 was found to recognize and bind to a cccDNA-like construct episomal DNA [12], which may be facilitated by PJA1 [15], an E3 ubiquitin ligase. cccDNA-bound Smc5/6 is recruited to promyelocytic leukemia (PML) bodies in the nucleus with the help of the SLF2 localization protein, another member of the Smc5/6 complex [16]. The localization of Smc5/6 to PML bodies may be important for the transcriptional repression of cccDNA since PML bodies (also known as nuclear domain 10 or ND10) are intra-nuclear complexes composed of numerous proteins that restrict transcription of many incoming DNA viruses [14]. The NSE2 protein of the Smc5/6 complex has also been implicated in mediating cccDNA silencing [17]. However, the exact mechanism underlying cccDNA silencing by Smc5/6 is yet to be fully characterized. Smc5/6 has been reported to repress transcription from the episomal genomes of other viruses, including HIV-1, by condensing their episomes into a tightly folded chromatin structure [10,18,19], which may be a similar mechanism to how it represses cccDNA.

To counteract the restrictive effect of Smc5/6, the HBx protein recruits cellular DNA damage-binding protein 1 (DDB1), which contains an E3 ubiquitin ligase that targets Smc5/6 for proteasomal degradation [12,13,14]. This antagonism of the Smc5/6 complex by HBx is an evolutionarily conserved function found in divergent mammalian HBV species [20] and other viruses restricted by Smc5/6, such as HIV-1, EBV, and KSHV, which each have a protein that targets the Smc5/6 complex for proteasomal degradation [11]. The therapeutic potential of silencing HBx was explored in an in vivo mouse model, where the silencing of HBV transcripts (including that of HBx) by siRNA and pegylated interferon-α (peg-IFNα), along with inhibition of viral re-entry, enabled the reappearance of Smc5/6, in turn suppressing cccDNA transcription. Conversely, the cessation of peg-IFNα led to the degradation of the re-emerged Smc5/6 [21].

### 3.2. TRIM Proteins

Tripartite motif (TRIM) proteins are a large and conserved family of over 80 known proteins that share a conserved N-terminal RING domain with E3 ubiquitin ligase activity and a variable C-terminal domain that binds to different proteins; in concert, the two domains promote the ubiquitination of bound proteins. Many TRIM proteins are induced by type I and II IFNs and can act as viral restriction factors by interacting with viral proteins or act indirectly by regulating the activity of other antiviral factors [22,23].

Many TRIM proteins have been found to inhibit the transcriptional activity of HBV either by affecting promoter activity or through the ubiquitination of HBV proteins. Zhang et al. [24] tested 38 human TRIM proteins by co-transfecting plasmids containing each of the TRIM proteins along with a plasmid containing the 1.3-fold HBV genome into HepG2 cells. They found that TRIM41 inhibited the activity of both the Enh I and EnhII/Core promoters (Cp). Further, the knockdown of TRIM41 in HepG2.2.15 cells increased levels of HBV preC/C RNA. TRIM25 [24] and TRIM22 [25] have also been reported to inhibit EnhII/Cp. Both the N-terminal RING domain and the C-terminal domain, which contains a nuclear localization signal, are important for the repression of cccDNA transcription by TRIM; however, the exact mechanism of transcriptional repression by these proteins is yet to be fully determined. TRIM56 indirectly inhibits Cp activity by targeting the inhibitor of nuclear factor kappa B (IκBα) for ubiquitination, inducing the phosphorylation and activation of NF-kB [26]. NF-kB activation is associated with diminished HBV transcription [27], likely via the upregulation of a plethora of antiviral genes.

TRIM proteins also inhibit HBV replication through the ubiquitination of various HBV proteins, targeting them for degradation. HBV Pol is targeted by TRIM21 [28], while HBx is targeted by TRIM26 [29], TRIM 21 [30], and TRIM28 [31]. TRIM38 also enhances the anti-HBV effect of IFN-α by promoting the expression of other antiviral proteins. Interestingly, TRIM38 levels were elevated in the PBMCs of early responders during peg-IFNα therapy in CHB patients [32], emphasizing its potential importance in vivo. In a novel therapeutic approach, a monoclonal antibody (mAb) against HBx was fused with a cell-penetrating peptide (Tat), allowing for the intracellular targeting of HBx. The mechanism that led to the suppression of HBV transcription was found to be TRIM21 dependent: TRIM21, which also functions as a cytosolic Fc receptor, bound to the fused anti-HBx mAb and mediated the degradation of HBx. TRIM21 also induced antiviral responses through the activation of NF-κB and IFN-β, leading to the suppression of HBV DNA and proteins in vitro and in a mouse model [33].

### 3.3. Transcriptional Silencing via the HBV Promoters

The Cp region regulates the transcription of the precore and pgRNA transcripts. Cp, in turn, is regulated by EnhII, located upstream of the Cp (overlapping with the HBx coding region) [34]. Several cellular transcription factors bind to the EnhII/Cp region to initiate or inhibit transcription [35]. In in vitro studies with hepatoma cell lines, the transcription factor Zinc finger E-box binding homeobox 2 (ZEB2) bound to Cp, and the overexpression of ZEB2 led to a reduction in HBV transcripts and secreted proteins [36]. Two other transcription factors, Maf bZIP transcription factor F (MafF) [37] and Homeobox protein MSX-1 (MSX1) [38], inhibit Cp activity by preventing the binding of a transcription enhancer, hepatocyte nuclear factor 4α (HNF-4α), to the EnhII/Cp region. The ISGs interferon-α inducible protein 6 (IFI6) [39] and IFI27 [40] have also been found to bind to the EnhII/Cp region, inhibiting HBV replication in vitro and in a mouse model. The transcription repressor, B cell lymphoma 6 (BCL6), was found to bind to all four HBV promoter sequences in an in vitro luciferase reporter gene assay and was reported to suppress HBV transcription in vitro and in a mouse model [41].

The zinc finger and homeoboxes 2 (ZHX2), a tumor suppressor, is also reported to repress HBV transcription by suppressing Cp, SPII (the promoter that regulates PreS2 and S transcription), and Xp (the promoter that regulates HBx transcription) promoters. In addition, ZHX2 has been described in vitro and in murine studies as a regulator of histone genes that exerts epigenetic repression of cccDNA transcription [42]. HBx, in turn, has been found to suppress ZHX2 expression through the activation of microRNA-155 (miR-155) [43]. Notably, hepatocyte nuclear factor 6 (HNF6) has also been found to repress the SPII promoter and accelerate pgRNA decay post-transcriptionally in the nucleus [44].

### 3.4. PRMT5

The protein arginine methyltransferase 5 (PRMT5) catalyzes symmetric dimethylation of arginine residues and participates in numerous cellular processes, including chromatin and transcription regulation [45]. PRMT5 has been found to preferentially silence cccDNA by symmetric dimethylation of arginine 3 on histone 4 (H4R3me2s) on cccDNA. In addition, PRMT5 was found to inhibit pgRNA encapsidation by binding to the RNase H domain of HBV Pol, interrupting its interaction with pgRNA [46].

## 4. Post-Transcriptional Processing and Regulation

Several host genes restrict HBV replication by inhibiting or degrading already formed HBV viral transcripts. Although not true inactivators of cccDNA transcription, restriction at these post-transcriptional steps in the lifecycle is usually evident as diminished intracellular viral transcripts despite the retention of cccDNA, which in experimental systems appears phenotypically similar to transcriptional silencing.

### 4.1. Mx Proteins

The Myxovirus resistance proteins (Mx) MxA (or MX1) and MxB (or MX2) are GTPases with antiviral activity against numerous viruses that are expressed in response to type I and III interferons. Both have an N-terminal GTPase domain, a middle domain, and a C-terminal GTPase effector domain. The Mx proteins can form dimers and oligomers to carry out their functions. Their intracellular localization may influence their function. MxA and a short isoform of MxB (76 kDa) are found in the cytoplasm [47], while the longer 78 kDa isoform of MxB can be found in the nucleus [48] and nuclear pore [49].

MxA reduced HBV protein secretion and DNA intermediates in studies with HBV and MxA co-transfected into hepatoma cell lines. The cytoplasmic localization of MxA is important for its anti-HBV activity [50]. Two mechanisms of HBV suppression by MxA have been proposed. First, MxA prevents the export of viral mRNAs [51], and second, MxA binds to the HBc protein and immobilizes it near the perinuclear membrane, thereby interrupting capsid formation [52]. Further studies are required to ascertain the exact mechanism of MxA restriction. Interestingly, HBV counters this restriction through HBc, which can suppress the MxA promoter [53,54,55].

MxB is also reported to decrease HBV RNA and cccDNA levels in HBV-transfected hepatoma cell lines and in infected primary human hepatocytes (PHH). The GTPase activity and oligomerization of MxB is important for its anti-HBV activity, which is speculated to be involved in inhibiting the conversion of rcDNA to cccDNA [56]. MxB is also known to inhibit the nuclear import of HIV-1 [57,58], which may be how it restricts HBV as well.

### 4.2. ZAP

Zinc finger antiviral protein (ZAP, also known as ZC3HAV1), an ISG [59], has two isoforms (ZAP-L and ZAP-S) and has antiviral activity against several RNA viruses. While ZAP does not have RNAse activity of its own, it functions by binding through its N-terminal to viral RNA motifs, designated as ZAP-responsive elements (ZRE), to recruit host RNA processing complexes that degrade viral RNA, potentially in concert with other ISGs [60]. Since pgRNA has a ZRE in the terminal region (nt 1820-1918), isoforms of ZAP may inhibit HBV replication via the post-transcriptional degradation of pgRNA. In a ZAP-transgenic mouse model transfected with an HBV-expressing plasmid, a significant decrease in HBV DNA replication intermediates and proteins was observed compared to non-transgenic control mice, although surprisingly only a modest decrease in HBV RNA was seen [61].

### 4.3. ISG20

Interferon-stimulated gene 20 kDA protein (ISG20) is a 3′ to 5′ exonuclease that cleaves single-stranded RNA and DNA, with a strong preference for RNA. It belongs to a family of 3′exonucleases that has three conserved exonuclease motifs named Exo I, Exo II, and Exo III. ISG20 interferes with the replication of many RNA viruses as well as Hepadnaviridae such as HBV [62]. ISG20 has been reported to inhibit HBV replication by the binding of its Exo III site to the 5′ (ε) stem loop region of pgRNA, promoting its degradation. The ε region is a unique structure of HBV required for genome packaging. The removal of four base pairs from the ε loop abrogated ISG20 activity [63]. It was further found that the methylation of A1907 (m6A) at the lower stem of the ε region of pgRNA is uniquely identified by an m6A reader protein, YTHDF2, suggesting that it is an important co-factor of the ISG20 recognition of pgRNA [64]. pgRNA on its own is insufficient to stimulate ISG20 upregulation, which generally requires type 1 interferon stimulation [65]. In another study, ISG20 was reported to inhibit HBV transcription and replication by directly binding to the EnhII/Cp region in an in vitro reporter assay [66].

Interestingly, ISG20 was also found to be important for cccDNA degradation after deamination by APOBEC3A (described below). ISG20 localized to the nuclei of IFNα-stimulated hepatocytes and was enriched on deoxyuridine-containing ssDNA, meant to model transcriptionally active and APOBEC3A-deaminated HBV DNA. ISG20 depletion attenuated the type I IFN-mediated cccDNA loss, while co-expression with APOBEC3A efficiently diminished cccDNA. Hence, ISG20 was proposed as an IFNα-induced nuclease that cleaves and degrades HBV cccDNA post-deamination by an APOBEC3 protein [67].

### 4.4. DEAD-Box Helicases

DEAD-box helicases derive their name from the conserved Asp-Glu-Ala-Asp (D-E-A-D) amino acid motif. They comprise a conserved family of proteins involved in RNA splicing, mRNA export, regulation of transcription and translation, and RNA decay. DEAD-box helicases are involved in a multitude of cellular processes, including innate immune signaling [68]. Several members of this family have been reported to sense HBV RNA, specifically the 5′-ε region of pgRNA, and either inhibit the subsequent steps of the interaction with HBV Pol (by DDX58 or RIG-I [5]), encapsidation (by DDX17 [69]), or reverse transcription (by DDX3 [70]). DDX3 was also found to suppress HBV transcription from cccDNA by an unelucidated mechanism and activate interferon regulatory factor (IRF) signaling [71]. Further, RIG-I, perhaps one of the best-characterized DEAD-box helicases, was reported to sense pgRNA and induce type I and III IFNs [5].

HBV has been reported to counter RIG-I activation by inducing microRNA-146a (miR-146a) expression, attenuating RIG-I expression in vitro. Silencing miR-146a in a mouse model of hydrodynamic infection was found to increase RIG-I expression and conversely decrease HBV DNA and proteins in mouse serum. Targeting miR-146a might be a therapeutic strategy to control HBV [72]. Additionally, a RIG-I agonist SB 9200 (Inarigivir) was found to be effective in the reduction of HBV DNA and proteins in a woodchuck CHB model [73]. However, the further investigation of Inarigivir was discontinued due to adverse effects in early human trials [74,75].

## 5. Viral Translation

### 5.1. Galectin-9

Galectins are an evolutionarily conserved family of glycan or carbohydrate-binding proteins with pleiotropic functions in immune responses. These proteins can be multi-valent and can form oligomers, activating distinct signaling pathways [76]. Galectin 9 (GAL9) specifically has been reported to restrict HBV replication by directing the autophagic degradation of HBc. The proposed mechanism is by viperin (an ISG), which promotes an interaction between GAL9 and HBc in the cytoplasm; the complex then associates with RING finger protein 13 (RNF13) to promote auto-ubiquitination, resulting in the recruitment of autophagy receptor p62 and the autophagosome machinery to degrade HBc [77].

### 5.2. Indoleamine-2, 3-Dioxygenase (IDO)

Indoleamine-2, 3-dioxygenase (IDO) is a cytosolic heme-containing enzyme that depletes tryptophan by catalyzing the oxidation of L-tryptophan into N-formylkynurenine [78]. Tryptophan is an essential amino acid; thus, its depletion is a defensive strategy utilized by mammalian host cells to inhibit intracellular pathogens [79]. IFN-γ-induced IDO expression inhibits HBV protein translation and HBV DNA replication without significantly altering cellular protein synthesis in vitro [80]. As determined in co-culture experiments with HBV-transfected Huh7 cells, IFN-γ produced by natural killer cells and plasmacytoid dendritic cell induced IDO activity in Huh7 cells, leading to HBV suppression. In humans, IDO activity is increased in acute self-resolving patients during peak ALT compared to people with CHB, people undergoing hepatic flares, and healthy controls, supporting a potential role of IDO as an anti-HBV effector [81].

## 6. Reverse Transcription

### 6.1. APOBEC3

Members of the evolutionarily conserved family of apolipoprotein B mRNA editing catalytic polypeptide-like (APOBEC) proteins bind and edit RNA and ssDNA by deaminating cytidine (C) to uracil (U). APOBECs have a broad range of functions, including in innate and adaptive immunity, development, evolution, and oncogenesis [82]. The interferon-inducible APOBEC3 subfamily consists of seven major homologs in humans (A3A-A3H): A3A, A3C, and A3H have a single cytosine deaminase (CD) domain. A3B, A3D, A3F, and A3G have two CD domains, of which only CD2 is catalytically active, while CD1 has roles in binding RNA or ssDNA and packaging APOBEC3 into viral capsids. The APOBEC3 enzymes especially target foreign RNA and DNA and inhibit the replication of a wide range of viruses using deaminase-dependent and deaminase-independent mechanisms [83]. The role of APOBEC3 as an antiviral restriction factor is perhaps best-described for HIV-1 [84,85,86]. The anti-HBV activity of the APOBEC3 proteins has been studied over the last two decades. APOBEC3 proteins (A3B, A3C, and A3G) can bind HBc and can be packaged in viral capsids [87,88]. They have been found to induce C to U mutations within viral capsids, starting from the reverse transcription start site and continuing through along the entire (-) DNA transcript. Different APOBEC3 proteins exhibit variable efficiencies of inducing hypermutations: A3B has the highest mutation rate (65%), followed by A3G, then A3H, and lastly A3C [89]. A3B, A3C, A3F, and A3G deaminate the minus strand of HBV, while A3B, A3F, and A3G also deaminate the plus strand [90]. Hypermutation due to cytosine deamination leads to lethal nascent viral genomes by adding premature stop codons and missense mutations in vital genes [91]. It is also suggested that the deaminated rcDNA can be degraded by cellular enzymes [67,92].

Deaminase-independent HBV restriction is also observed, especially for A3G. A3G causes significant inhibition of HBV DNA and protein production in hepatoma cells co-transfected with HBV and A3G [93,94]; however, deamination-inactive A3G mutants are also able to reduce HBV replication almost as well as the wildtype A3G [95]. The inhibitory effect of A3G is in the early stages of the reverse transcription, specifically during minus strand synthesis [96]. Although the exact mechanism of the deaminase-independent inhibition of HBV replication by A3G is not yet known, various models have been proposed from studies in HIV-1 [97].

A3B, A3C, A3F, and A3G are expressed in the liver and, as noted above, can be induced by IFN-α [98]. The expression of A3A has been reported to be induced by IFN-α [99] and IFN-γ and of A3B by IFN-γ and TNF-α. A3A and A3B have also been found to be elevated in acute HBV infection compared to CHB and healthy control biopsy samples, perhaps due to the elevated IFN-γ and TNF-α found in serum [100].

There is interest in harnessing the anti-HBV activity of APOBEC3 proteins therapeutically, as has been done by the transient activation of APOBEC3 genes A3A, A3B, A3G, and AID by implementing a CRISPR activation-based approach (CRISPRa) [101]. Although this approach effectively reduced HBV replication in phases of infection where there is high viral replication, further research is required to quantify and reduce the off-target effects of hypermutation in low viral replication cells [102].

### 6.2. SAMHD1

Deoxynucleoside triphosphates (dNTPs) are the building blocks of cellular genomic material: they are biosynthesized or broken down as per the metabolic need of the cells. The sterile alpha motif (SAM) domain and histidine aspartate (HD) domain-containing protein 1 (SAMHD1) is a negative regulator of the cellular dNTP pool. In its active form, SAMHD1 degrades dNTPs into 2′-deoxynucleoside and triphosphate [103]. In addition to host genome maintenance, dNTPs are also required for the genome replication of viruses. SAMHD1 has been found to be a potent intrinsic immune restriction factor that attenuates the replication of several DNA and retroviruses (including HIV-1) [104] and is also found to be upregulated by type I and II interferons in hepatic cells [105,106]. The exogenous expression of SAMHD1 was found to impede HBV replication in two different hepatic cells lines [105]. The dNTPase activity of SAMHD1 reduced levels of extracellular viral DNA (largely rcDNA) and intracellular reverse transcription products but not cccDNA or HBV RNA, while the addition of dNTPs rescued viral replication [106,107]. Interestingly, HBV was found to counter the restriction of SAMHD1 by inducing dNTP synthesis in hepatoma cells through the activation of the host R2 gene, a key component of ribonucleotide reductase that catalyzes the formation of dNTPs [108,109].

## 7. Secretion

### 7.1. Tetherin

Tetherin, also known as BST2, is a glycoprotein with unique topology: it has an N-terminal cytoplasmic tail, a transmembrane domain, a coiled-coil ectodomain, and lastly a C-terminal glycosylphosphatidylinositol (GPI) anchor. This doubly anchored molecule is mainly localized to apical membranes and to some extent in the trans-Golgi network (TGN). Tetherin, an ISG, forms homo-dimers that inhibit the budding of several enveloped viruses, including HIV-1 [110]. Budding viral particles that include the insertion of the C-terminal of tetherin remain anchored to the host cell. In addition, the cytoplasmic N-terminus can initiate intracellular signaling cascades to induce cytokine and chemokine expression [111].

Tetherin has been reported to selectively inhibit the secretion of enveloped HBV virions in vitro. Tetherin and HBV can be found co-localized in intracellular multivesicular bodies (MVBs) of cells. It is speculated that the intracellular environment of hepatocytes is not conducive for optimal tetherin activity [112,113]. Moreover, Sm-HBs can attenuate tetherin function by inhibiting its dimerization [114].

### 7.2. SERINC5

Serine incorporator 5 (SERINC5) protein belongs to a family of proteins involved in the incorporation of the amino acid serine into membranes to facilitate the biosynthesis of phosphatidylserine and sphingolipids [115]. SERINC5 may inhibit HBV virion secretion by specifically interacting with the L-, M-, and Sm-HBs proteins in the TGN and interfering with their glycosylation, which inhibits their secretion from infected cells [116].

## 8. Discussion

In adults, 90% of acute HBV infections spontaneously resolve: it is tempting to consider whether this involves the recruitment of host restriction factors. Chronic infection has several phases of progressive HBV control during which restriction factors may play a role. In addition, since many of the restriction factors are ISGs, they are likely to be induced in response to peg-IFN-α treatment during CHB, although only ~30% of recipients respond to this treatment. This may be due to the limitations of the amount of peg-IFN-α that can be tolerated. Questions remain as to whether host restriction factors play a role in the natural or therapeutic control of HBV and whether intrinsic differences in their expression or activity contribute to the variability in this control.

The importance of these host restriction factors may be inferred from the countermeasures HBV employs to maintain its replication (Table 1). As noted above, HBx has been found to target restriction factors such as Smc5/6 and ZEB2 for degradation [117]. A reciprocal relationship may be found for many of the factors such that when HBV replication is abundant, host restriction factors are repressed. Logically, when treatment reduces HBV replication, restriction factors may be derepressed, exerting additive inhibition of HBV.

The abundance of restriction factors directed at suppressing cccDNA transcription underscores the importance of this step in the lifecycle and may offer clues as to how to convert transcriptionally active cccDNA into inactive cccDNA, an important step in achieving a functional cure. We and others have found that the treatment of CHB with nucleos(t)ide analogues (NUCs) that interrupt reverse transcription is associated with reduced cccDNA transcriptional activity [118,119,120,121,122]. While this apparent transcriptional silencing of cccDNA is not predicted by the actions of NUCs, it is possible that host restriction factors are somehow activated during NUCs to suppress or degrade viral transcripts.

A common limitation of studies of the host restriction of HBV is that many of the factors were discovered and characterized in vitro in overexpression systems with co-transfection of an HBV-expressing plasmid. In vivo studies in mice may be confounding since some of these factors function differently in humans (e.g., Mx proteins). With the advent of the HBV culture system, it would be important to validate the potency of these restriction factors in an infection model. Similarly, only a handful of these factors have been observed and linked to HBV control in human liver tissues. Thus, it is critical to characterize the expression and activity of these factors in people with CHB. Notably, there is comparatively little known about the intracellular restriction of the initial steps of the HBV infection, including entry, uncoating, and nuclear import. More research is required to interrogate these key steps in the HBV lifecycle.

It is promising to consider therapeutic strategies that enhance the endogenous expression of select restriction factors to facilitate HBV control and even a cure. Several have been investigated, although a clinically safe and effective therapeutic is yet to emerge. Perhaps most relevant are siRNA and antisense RNAs presently in clinical trials that are directed towards HBV S transcripts, which encode HBsAg, that have had some success in suppressing HBsAg levels [123,124]. Because all HBV transcripts overlap at their 3′ end, these RNA interference strategies also target the short HBx gene products. Since, as described above, HBx is a potent transcriptional activator that leads to the degradation of Smc5/6 and other restriction factors, it is tantalizing to consider that the success of these siRNAs/antisense RNAs is at least partly due to the rescue of Smc5/6 levels that further suppress cccDNA transcription. Indeed, it is important to note that recent trials of siRNAs have all benefitted from the addition of immune modulators, most commonly peg-IFN-α. Even bepirovirsen, a leading antisense RNA candidate for HBV, which did not explicitly benefit from additional peg-IFN-α, has been found to have intrinsic immunostimulatory activity [125,126]. Systemic peg-IFN-α clearly upregulates several of the ISGs that are also HBV restriction factors, described above. It is notable that peg-IFN-α has a higher rate of HBsAg clearance, i.e., a functional cure, compared to NUCs, the mainstay of CHB treatment, underscoring the potency of the suite of restriction factors described here. Novel approaches are also being considered. As noted above, the gene-editing revolution, heralded by the advent of CRISPR technologies, has been applied to specifically and transiently activate APOBEC genes to target HBV in vitro. If promising, this approach could be employed to activate other restriction factors or even collectively to activate a suite of restriction factors. Thus, despite the inherent challenges, the development of strategies to enhance endogenous anti-HBV activity is warranted.

## 9. Conclusions

The literature regarding the roles and mechanisms of different restriction factors is still evolving. Host restriction factors clearly play important roles as effectors of antiviral responses by the host cell against HBV infection in model systems (Table 1). To understand their clinical relevance requires carefully designed studies in people with HBV infection and in representative animal models and in culture systems that explore the dependence of HBV control on these factors. A better understanding of the mechanisms by which host factors restrict HBV may facilitate their development as potential therapeutics to achieve a functional cure.

**Table 1:** Host factors that restrict HBV replication, their mechanism of HBV restriction, and mechanisms employed by HBV to evade the restriction.

Factor	Mechanism of HBV Restriction	Viral Counter-Action
Transcription		
Smc5/6	Inactivates cccDNA [12,13,14,15,16,17]	HBx-mediated ubiquitination of Smc5/6 [12,13,14]
TRIM proteins	(1) Represses EnhII/Core promoter—TRIM41 [24], TRIM22 [25], TRIM25 [24], TRIM 56 [26]; (2) Ubiquitinates of HBV proteins: Pol by TRIM21 [28]; HBx by TRIM26 [29], TRIM21 [30], TRIM28 [31].	
ZEB2, MafF, MSX1, IFI6, IFI27, BCL6, ZHX2, HNF6	(1) ZEB2, MafF, MSX1, IFI5, IFI27 bind and suppress the Core Promoter (Cp) [36,37,38,39,40]; (2) BCL6 is reported to bind and suppress all 4 HBV promoters [41]; (3) ZHX2 represses Cp, SPII and X [42]; (4) HNF6 represses SPII [44].	(1) HBx-mediated ubiquitination of ZEB2 [111]; (2) HBx activates miRNA 155 to suppress ZHX2 [43].
PRMT5	(1) cccDNA inactivation by H2R3me2s dimethylation; (2) Inhibits encapsidation [46].	
Post-transcriptional processing and regulation		
Mx proteins	(1) MxA: speculated to be either by preventing export of HBV mRNAs [51] or by immobilizing HBc near the peri-nuclear membrane [52]; (2) MxB: speculated to inhibit conversion of rcDNA to cccDNA [56].	HBc can inhibit the MxA promoter [53,54,55]
ZAP	Binds to ZRE (ZAP responsive elements) region on pgRNA; may recruit other host proteins to degrade pgRNA [59]	
ISG20	(1) Binds to 5′ (ε) stem loop region of pgRNA and degrades it through its exonuclease activity [61]; (2) Binds the HBV EnhII/Cp region and inhibits its activity [64]; (3) Possibly degrades deaminated HBV DNA (deaminated by APOBEC3) [65].	
DEAD/H-box helicases	Binds to 5′ (ε) stem loop region of pgRNA and inhibits (a) interaction with polymerase (by RIG-I) [5]; (b) encapsidation (by DDX17) [67]; (c) reverse transcription (by DDX3) [68]; (d) activates interferon stimulating pathways (DDX3 and RIG-I) [5,69].	HBV induces miR146a, which suppresses RIG-I expression [70]
Viral translation		
Galectin-9	Directs autophagic degradation of HBc [75]	
Indoleamine-2, 3-dioxygenase (IDO)	Depletes tryptophan, hampering viral translation [78]	
Reverse transcription		
APOBEC3	(1) Introduces C to U mutations during reverse transcription [82,83,84,85]; (2) Deaminase-independent mechanisms (by A3G) not fully elucidated yet [90,91].	
SAMHD1	Depletes dNTP pool from the cell, hindering synthesis of new HBV DNA genomes [100,101,102]	HBV can induce dNTP synthesis [103,104]
Secretion		
Tetherin	Inhibits secretion of enveloped HBV virions from the trans-golgi network (TGN) [106,107]	Sm-HBs can inhibit tetherin dimerization [108]
SERINC5	Interferes with glycosylation of S-proteins, hindering their secretion from the TGN [110]	

## References

[1] Mueller S.N., Rouse B.T. (2013). Host defenses to viruses. Clin. Immunol..

[2] Dunn C., Peppa D., Khanna P., Nebbia G., Jones M., Brendish N., Lascar R.M., Brown D., Gilson R.J., Tedder R.J. (2009). Temporal Analysis of Early Immune Responses in Patients With Acute Hepatitis B Virus Infection. Gastroenterology.

[3] Suslov A., Boldanova T., Wang X., Wieland S., Heim M.H. (2018). Hepatitis B Virus Does Not Interfere With Innate Immune Responses in the Human Liver. Gastroenterology.

[4] Zhang Z., Trippler M., Real C.I., Werner M., Luo X., Schefczyk S., Kemper T., Anastasiou O.E., Ladiges Y., Treckmann J. (2020). Hepatitis B Virus Particles Activate Toll-Like Receptor 2 Signaling Initially Upon Infection of Primary Human Hepatocytes. Hepatology.

[5] Sato S., Li K., Kameyama T., Hayashi T., Ishida Y., Murakami S., Watanabe T., Iijima S., Sakurai Y., Watashi K. (2014). The RNA Sensor RIG-I Dually Functions as an Innate Sensor and Direct Antiviral Factor for Hepatitis B Virus. Immunity.

[6] Yang Y., Zhao X., Wang Z., Shu W., Li L., Li Y., Guo Z., Gao B., Xiong S. (2020). Nuclear Sensor Interferon-Inducible Protein 16 Inhibits the Function of Hepatitis B Virus Covalently Closed Circular DNA by Integrating Innate Immune Activation and Epigenetic Suppression. Hepatology.

[7] Tsukuda S., Watashi K. (2020). Hepatitis B virus biology and life cycle. Antivir. Res..

[8] Gutierrez-Escribano P., Hormeno S., Madariaga-Marcos J., Sole-Soler R., O’Reilly F.J., Morris K., Aicart-Ramos C., Aramayo R., Montoya A., Kramer H. (2020). Purified Smc5/6 Complex Exhibits DNA Substrate Recognition and Compaction. Mol. Cell.

[9] Serrano D., Cordero G., Kawamura R., Sverzhinsky A., Sarker M., Roy S., Malo C., Pascal J.M., Marko J.F., D’Amours D. (2020). The Smc5/6 Core Complex Is a Structure-Specific DNA Binding and Compacting Machine. Mol. Cell.

[10] Roy S., Adhikary H., D’Amours D. (2024). The SMC5/6 complex: Folding chromosomes back into shape when genomes take a break. Nucleic Acids Res..

[11] Irwan I.D., Cullen B.R. (2023). The SMC5/6 complex: An emerging antiviral restriction factor that can silence episomal DNA. PLoS Pathog..

[12] Decorsiere A., Mueller H., van Breugel P.C., Abdul F., Gerossier L., Beran R.K., Livingston C.M., Niu C., Fletcher S.P., Hantz O. (2016). Hepatitis B virus X protein identifies the Smc5/6 complex as a host restriction factor. Nature.

[13] Murphy C.M., Xu Y., Li F., Nio K., Reszka-Blanco N., Li X., Wu Y., Yu Y., Xiong Y., Su L. (2016). Hepatitis B Virus X Protein Promotes Degradation of SMC5/6 to Enhance HBV Replication. Cell Rep..

[14] Niu C., Livingston C.M., Li L., Beran R.K., Daffis S., Ramakrishnan D., Burdette D., Peiser L., Salas E., Ramos H. (2017). The Smc5/6 Complex Restricts HBV when Localized to ND10 without Inducing an Innate Immune Response and Is Counteracted by the HBV X Protein Shortly after Infection. PLoS ONE.

[15] Xu W., Ma C., Zhang Q., Zhao R., Hu D., Zhang X., Chen J., Liu F., Wu K., Liu Y. (2018). PJA1 Coordinates with the SMC5/6 Complex To Restrict DNA Viruses and Episomal Genes in an Interferon-Independent Manner. J. Virol..

[16] Yao Q., Peng B., Li C., Li X., Chen M., Zhou Z., Tang D., He J., Wu Y., Sun Y. (2023). SLF2 Interacts with the SMC5/6 Complex to Direct Hepatitis B Virus Episomal DNA to Promyelocytic Leukemia Bodies for Transcriptional Repression. J. Virol..

[17] Abdul F., Diman A., Baechler B., Ramakrishnan D., Kornyeyev D., Beran R.K., Fletcher S.P., Strubin M. (2022). Smc5/6 silences episomal transcription by a three-step function. Nat. Struct. Mol. Biol..

[18] Han C., Zhang D., Gui C., Huang L., Chang S., Dong L., Bai L., Wu S., Lan K. (2022). KSHV RTA antagonizes SMC5/6 complex-induced viral chromatin compaction by hijacking the ubiquitin-proteasome system. PLoS Pathog..

[19] Dupont L., Bloor S., Williamson J.C., Cuesta S.M., Shah R., Teixeira-Silva A., Naamati A., Greenwood E.J.D., Sarafianos S.G., Matheson N.J. (2021). The SMC5/6 complex compacts and silences unintegrated HIV-1 DNA and is antagonized by Vpr. Cell Host Microbe.

[20] Abdul F., Filleton F., Gerossier L., Paturel A., Hall J., Strubin M., Etienne L. (2018). Smc5/6 Antagonism by HBx Is an Evolutionarily Conserved Function of Hepatitis B Virus Infection in Mammals. J. Virol..

[21] Allweiss L., Giersch K., Pirosu A., Volz T., Muench R.C., Beran R.K., Urban S., Javanbakht H., Fletcher S.P., Lutgehetmann M. (2022). Therapeutic shutdown of HBV transcripts promotes reappearance of the SMC5/6 complex and silencing of the viral genome in vivo. Gut.

[22] van Gent M., Sparrer K.M.J., Gack M.U. (2018). TRIM Proteins and Their Roles in Antiviral Host Defenses. Annu. Rev. Virol..

[23] Giraldo M., Hage A., van Tol S., Rajsbaum R. (2020). TRIM Proteins in Host Defense and Viral Pathogenesis. Curr. Clin. Microbiol. Rep..

[24] Zhang S., Guo J.-T., Wu J.Z., Yang G. (2013). Identification and Characterization of Multiple TRIM Proteins That Inhibit Hepatitis B Virus Transcription. PLoS ONE.

[25] Gao B., Duan Z., Xu W., Xiong S. (2009). Tripartite motif-containing 22 inhibits the activity of hepatitis B virus core promoter, which is dependent on nuclear-located RING domain. Hepatology.

[26] Tian X., Dong H., Lai X., Ou G., Cao J., Shi J., Xiang C., Wang L., Zhang X., Zhang K. (2022). TRIM56 impairs HBV infection and replication by inhibiting HBV core promoter activity. Antivir. Res..

[27] Lin Y.C., Hsu E.C., Ting L.P. (2009). Repression of hepatitis B viral gene expression by transcription factor nuclear factor-kappaB. Cell. Microbiol..

[28] Mu T., Zhao X., Zhu Y., Fan H., Tang H. (2020). The E3 Ubiquitin Ligase TRIM21 Promotes HBV DNA Polymerase Degrada-tion. Viruses.

[29] Luo M., Hou J., Mai H., Chen J., Chen H., Zhou B., Hou J., Jiang D.K. (2022). TRIM26 inhibits hepatitis B virus repli-cation by promoting HBx degradation and TRIM26 genetic polymorphism predicts PegIFNalpha treatment response of HBeAg-positive chronic hepatitis B Patients. Aliment. Pharmacol. Ther..

[30] Song Y., Li M., Wang Y., Zhang H., Wei L., Xu W. (2021). E3 ubiquitin ligase TRIM21 restricts hepatitis B virus replication by targeting HBx for proteasomal degradation. Antivir. Res..

[31] Liu C., Zhao K., Chen Y., Yao Y., Tang J., Wang J., Xu C., Yang Q., Zheng Y., Yuan Y. (2023). Mitochondrial Glycerol-3-Phosphate Dehydrogenase Restricts HBV Replication via the TRIM28-Mediated Degradation of HBx. J. Virol..

[32] Luo H., Hu X., Li Y., Lei D., Tan G., Zeng Y., Qin B. (2023). The antiviral activity of tripartite motif protein 38 in hepatitis B virus replication and gene expression and its association with treatment responses during PEG-IFN-α antiviral therapy. Virology.

[33] Zhang J.-F., Xiong H.-L., Cao J.-L., Wang S.-J., Guo X.-R., Lin B.-Y., Zhang Y., Zhao J.-H., Wang Y.-B., Zhang T.-Y. (2018). A cell-penetrating whole molecule antibody targeting intracellular HBx suppresses hepatitis B virus via TRIM21-dependent pathway. Theranostics.

[34] Quarleri J. (2014). Core promoter: A critical region where the hepatitis B virus makes decisions. World J. Gastroenterol..

[35] Turton K.L., Meier-Stephenson V., Badmalia M.D., Coffin C.S., Patel T.R. (2020). Host Transcription Factors in Hepatitis B Virus RNA Synthesis. Viruses.

[36] He Q., Li W., Ren J., Huang Y., Huang Y., Hu Q., Chen J., Chen W. (2016). ZEB2 inhibits HBV transcription and repli-cation by targeting its core promoter. Oncotarget.

[37] Ibrahim M.K., Abdelhafez T.H., Takeuchi J.S., Wakae K., Sugiyama M., Tsuge M., Ito M., Watashi K., El Kassas M., Kato T. (2021). MafF Is an Antiviral Host Factor That Suppresses Transcription from Hepatitis B Virus Core Promoter. J. Virol..

[38] Shen Z., Zhang S., Gao Z., Yu X., Wang J., Pan S., Kang N., Liu N., Xu H., Liu M. (2024). Intrahepatic homeobox protein MSX-1 is a novel host restriction factor of hepatitis B virus. J. Virol..

[39] Sajid M., Ullah H., Yan K., He M., Feng J., Shereen M.A., Hao R., Li Q., Guo D., Chen Y. (2021). The Functional and Antiviral Activity of Interferon Alpha-Inducible IFI6 Against Hepatitis B Virus Replication and Gene Expression. Front. Immunol..

[40] Ullah H., Sajid M., Yan K., Feng J., He M., Shereen M.A., Li Q., Xu T., Hao R., Guo D. (2021). Antiviral Activity of Interferon Alpha-Inducible Protein 27 Against Hepatitis B Virus Gene Expression and Rep-lication. Front. Microbiol..

[41] Lin C.-T., Hsieh Y.-T., Yang Y.-J., Chen S.-H., Wu C.-H., Hwang L.-H. (2019). B-Cell Lymphoma 6 (BCL6) Is a Host Restriction Factor That Can Suppress HBV Gene Expression and Modulate Immune Responses. Front. Microbiol..

[42] Xu L., Wu Z., Tan S., Wang Z., Lin Q., Li X., Song X., Liu Y., Song Y., Zhang J. (2018). Tumor suppressor ZHX2 restricts hepatitis B virus replication via epigenetic and non-epigenetic manners. Antivir. Res..

[43] Song X., Tan S., Wu Z., Xu L., Wang Z., Xu Y., Wang T., Gao C., Gong Y., Liang X. (2018). HBV suppresses ZHX2 expression to promote proliferation of HCC through miR-155 activation. Int. J. Cancer.

[44] Hao R., He J., Liu X., Gao G., Liu D., Cui L., Yu G., Yu W., Chen Y., Guo D. (2015). Inhibition of Hepatitis B Virus Gene Expression and Replication by Hepatocyte Nuclear Factor 6. J. Virol..

[45] Lubyova B., Hodek J., Zabransky A., Prouzova H., Hubalek M., Hirsch I., Weber J. (2017). PRMT5: A novel regulator of Hepatitis B virus replication and an arginine methylase of HBV core. PLoS ONE.

[46] Zhang W., Chen J., Wu M., Zhang X., Zhang M., Yue L., Li Y., Liu J., Li B., Shen F. (2017). PRMT5 restricts hepatitis B virus replication through epigenetic repression of covalently closed circular DNA transcription and interference with pregenomic RNA encapsidation. Hepatology.

[47] Haller O., Staeheli P., Schwemmle M., Kochs G. (2015). Mx GTPases: Dynamin-like antiviral machines of innate immunity. Trends Microbiol..

[48] Melén K., Keskinen P., Ronni T., Sareneva T., Lounatmaa K., Julkunen I. (1996). Human MxB Protein, an Interferon-α-inducible GTPase, Contains a Nuclear Targeting Signal and Is Localized in the Heterochromatin Region beneath the Nuclear Envelope. J. Biol. Chem..

[49] King M.C., Raposo G., Lemmon M.A. (2004). Inhibition of nuclear import and cell-cycle progression by mutated forms of the dynamin-like GTPase MxB. Proc. Natl. Acad. Sci. USA.

[50] Yu Z., Wang Z., Chen J., Li H., Lin Z., Zhang F., Zhou Y., Hou J. (2008). GTPase activity is not essential for the inter-feron-inducible MxA protein to inhibit the replication of hepatitis B virus. Arch. Virol..

[51] Gordien E., Rosmorduc O., Peltekian C., Garreau F., Brechot C., Kremsdorf D. (2001). Inhibition of hepatitis B virus repli-cation by the interferon-inducible MxA protein. J. Virol..

[52] Li N., Zhang L., Chen L., Feng W., Xu Y., Chen F., Liu X., Chen Z., Liu W. (2012). MxA inhibits hepatitis B virus replication by interaction with hepatitis B core antigen. Hepatology.

[53] Rosmorduc O., Sirma H., Soussan P., Gordien E., Lebon P., Horisberger M., Br Chot C., Kremsdorf D. (1999). Inhibition of interferon-inducible MxA protein expression by hepatitis B virus capsid protein. J. Gen. Virol..

[54] Fernández M., Quiroga J.A., Martín J., Pardo M., Horisberger M.A., Carreño V. (1997). Impaired interferon induction of human MxA protein in chronic hepatitis B virus infection. J. Med. Virol..

[55] Zhijian Y., Zhen H., Fan Z., Jin Y., Qiwen D., Zhongming Z. (2010). Hepatitis B virus core protein with hot-spot mutations inhibit MxA gene transcription but has no effect on inhibition of virus replication by interferon α. Virol. J..

[56] Wang Y.-X., Niklasch M., Liu T., Wang Y., Shi B., Yuan W., Baumert T.F., Yuan Z., Tong S., Nassal M. (2019). Interferon-inducible MX2 is a host restriction factor of hepatitis B virus replication. J. Hepatol..

[57] Fricke T., White T.E., Schulte B., De Souza Aranha Vieira D.A., Dharan A., Campbell E.M., Brandariz-Nuñez A., Diaz-Griffero F. (2014). MxB binds to the HIV-1 core and prevents the uncoating process of HIV-1. Retrovirology.

[58] Dicks M.D.J., Betancor G., Jimenez-Guardeño J.M., Pessel-Vivares L., Apolonia L., Goujon C., Malim M.H. (2018). Multiple components of the nuclear pore complex interact with the amino-terminus of MX2 to facilitate HIV-1 restriction. PLoS Pathog..

[59] Mao R., Nie H., Cai D., Zhang J., Liu H., Yan R., Cuconati A., Block T.M., Guo J.-T., Guo H. (2013). Inhibition of Hepatitis B Virus Replication by the Host Zinc Finger Antiviral Protein. PLoS Pathog..

[60] Guo X., Carroll J.-W.N., MacDonald M.R., Goff S.P., Gao G. (2004). The Zinc Finger Antiviral Protein Directly Binds to Specific Viral mRNAs through the CCCH Zinc Finger Motifs. J. Virol..

[61] Chen E.-Q., Dai J., Bai L., Tang H. (2015). The efficacy of zinc finger antiviral protein against hepatitis B virus transcription and replication in tansgenic mouse model. Virol. J..

[62] Deymier S., Louvat C., Fiorini F., Cimarelli A. (2022). ISG20: An enigmatic antiviral RNase targeting multiple viruses. FEBS Open Bio.

[63] Liu Y., Nie H., Mao R., Mitra B., Cai D., Yan R., Guo J.-T., Block T.M., Mechti N., Guo H. (2017). Interferon-inducible ribonuclease ISG20 inhibits hepatitis B virus replication through directly binding to the epsilon stem-loop structure of viral RNA. PLoS Pathog..

[64] Imam H., Kim G.-W., Mir S.A., Khan M., Siddiqui A. (2020). Interferon-stimulated gene 20 (ISG20) selectively degrades N6-methyladenosine modified Hepatitis B Virus transcripts. PLoS Pathog..

[65] Leong C.R., Funami K., Oshiumi H., Mengao D., Takaki H., Matsumoto M., Aly H.H., Watashi K., Chayama K., Seya T. (2016). Interferon-stimulated gene of 20 kDa protein (ISG20) degrades RNA of hepatitis B virus to impede the replication of HBV in vitro and in vivo. Oncotarget.

[66] Park Y.K., Lee S.Y., Lee A.R., Kim K.C., Kim K., Kim K.H., Choi B.S. (2020). Antiviral activity of interferon-stimulated gene 20, as a putative repressor binding to hepatitis B virus enhancer II and core promoter. J. Gastroenterol. Hepatol..

[67] Stadler D., Kaechele M., Jones A.N., Hess J., Urban C., Schneider J., Xia Y., Oswald A., Nebioglu F., Bester R. (2021). Interferon-induced degradation of the persistent hepatitis B virus cccDNA form depends on ISG20. Embo Rep..

[68] You H., Ma L., Wang X., Zhang F., Han Y., Yao J., Pan X., Zheng K., Kong F., Tang R. (2022). The emerging role of DEAD/H-box helicases in hepatitis B virus infection. Front. Cell. Infect. Microbiol..

[69] Mao R., Dong M., Shen Z., Zhang H., Liu Y., Cai D., Mitra B., Zhang J., Guo H. (2021). RNA Helicase DDX17 Inhibits Hepatitis B Virus Replication by Blocking Viral Pregenomic RNA Encapsidation. J. Virol..

[70] Wang H., Kim S., Ryu W.-S. (2009). DDX3 DEAD-Box RNA Helicase Inhibits Hepatitis B Virus Reverse Transcription by Incorporation into Nucleocapsids. J. Virol..

[71] Ko C., Lee S., Windisch M.P., Ryu W.-S. (2014). DDX3 DEAD-Box RNA Helicase Is a Host Factor That Restricts Hepatitis B Virus Replication at the Transcriptional Level. J. Virol..

[72] Hou Z., Zhang J., Han Q., Su C., Qu J., Xu D., Zhang C., Tian Z. (2016). Hepatitis B virus inhibits intrinsic RIG-I and RIG-G immune signaling via inducing miR146a. Sci. Rep..

[73] Korolowicz K.E., Iyer R.P., Czerwinski S., Suresh M., Yang J., Padmanabhan S., Sheri A., Pandey R.K., Skell J., Marquis J.K. (2016). Antiviral Efficacy and Host Innate Immunity Associated with SB 9200 Treatment in the Woodchuck Model of Chronic Hepatitis B. PLoS ONE.

[74] Agarwal K., Afdhal N., Coffin C., Fung S., Dusheiko G., Foster G., Elkhashab M., Tam E., Ramji A., Iyer R. (2020). Liver toxicity in the Phase 2 Catalyst 206 trial of Inarigivir 400 mg daily added to a nucleoside in HBV EAg negative patients. J. Hepatol..

[75] Yuen M.F., Chen C.Y., Liu C.J., Jeng W.J., Elkhashab M., Coffin C.S., Kim W., Greenbloom S., Ramji A., Lim Y.S. (2023). A phase 2, open-label, randomized, multiple-dose study evaluating Inarigivir in treatment-naive patients with chronic hepatitis B. Liver Int..

[76] Rabinovich G.A., Toscano M.A. (2009). Turning ‘sweet’ on immunity: Galectin-glycan interactions in immune tolerance and in-flammation. Nat. Rev. Immunol..

[77] Miyakawa K., Nishi M., Ogawa M., Matsunaga S., Sugiyama M., Nishitsuji H., Kimura H., Ohnishi M., Wa-tashi K., Shimotohno K. (2022). Galectin-9 restricts hepatitis B virus replication via p62/SQSTM1-mediated selective autophagy of viral core proteins. Nat. Commun..

[78] Thackray S.J., Mowat C.G., Chapman S.K. (2008). Exploring the mechanism of tryptophan 2,3-dioxygenase. Biochem. Soc. Trans..

[79] Ren W., Rajendran R., Zhao Y., Tan B., Wu G., Bazer F.W., Zhu G., Peng Y., Huang X., Deng J. (2018). Amino Acids As Mediators of Metabolic Cross Talk between Host and Pathogen. Front. Immunol..

[80] Mao R., Zhang J., Jiang D., Cai D., Levy J.M., Cuconati A., Block T.M., Guo J.-T., Guo H. (2011). Indoleamine 2,3-Dioxygenase Mediates the Antiviral Effect of Gamma Interferon against Hepatitis B Virus in Human Hepatocyte-Derived Cells. J. Virol..

[81] Yoshio S., Sugiyama M., Shoji H., Mano Y., Mita E., Okamoto T., Matsuura Y., Okuno A., Takikawa O., Mizokami M. (2016). Indoleamine-2,3-dioxygenase as an effector and an indicator of protective immune responses in pa-tients with acute hepatitis B. Hepatology.

[82] Salter J.D., Bennett R.P., Smith H.C. (2016). The APOBEC Protein Family: United by Structure, Divergent in Function. Trends Biochem. Sci..

[83] Sadeghpour S., Khodaee S., Rahnama M., Rahimi H., Ebrahimi D. (2021). Human APOBEC3 Variations and Viral Infection. Viruses.

[84] Sheehy A.M., Gaddis N.C., Choi J.D., Malim M.H. (2002). Isolation of a human gene that inhibits HIV-1 infection and is suppressed by the viral Vif protein. Nature.

[85] Zhang H., Yang B., Pomerantz R.J., Zhang C., Arunachalam S.C., Gao L. (2003). The cytidine deaminase CEM15 induces hypermutation in newly synthesized HIV-1 DNA. Nature.

[86] Malim M.H. (2008). APOBEC proteins and intrinsic resistance to HIV-1 infection. Philos. Trans. R. Soc. B Biol. Sci..

[87] Baumert T.F., Rösler C., Malim M.H., von Weizsäcker F. (2007). Hepatitis B virus DNA is subject to extensive editing by the human deaminase APOBEC3C. Hepatology.

[88] Chen Y., Hu J., Cai X., Huang Y., Zhou X., Tu Z., Hu J., Tavis J.E., Tang N., Huang A. (2017). APOBEC3B edits HBV DNA and inhibits HBV replication during reverse transcription. Antivir. Res..

[89] Chen Z., Eggerman T.L., Bocharov A.V., Baranova I.N., Vishnyakova T.G., Patterson A.P. (2021). APOBEC3-induced mutation of the hepatitis virus B DNA genome occurs during its viral RNA reverse transcription into (−)-DNA. J. Biol. Chem..

[90] Suspène R., Guétard D., Henry M., Sommer P., Wain-Hobson S., Vartanian J.-P. (2005). Extensive editing of both hepatitis B virus DNA strands by APOBEC3 cytidine deaminases in vitro and in vivo. Proc. Natl. Acad. Sci. USA.

[91] Stavrou S., Ross S.R. (2015). APOBEC3 Proteins in Viral Immunity. J. Immunol..

[92] Stenglein M.D., Burns M.B., Li M., Lengyel J., Harris R.S. (2010). APOBEC3 proteins mediate the clearance of foreign DNA from human cells. Nat. Struct. Mol. Biol..

[93] Turelli P., Mangeat B., Jost S., Vianin S., Trono D. (2004). Inhibition of Hepatitis B Virus Replication by APOBEC3G. Science.

[94] Lei Y.C., Hao Y.H., Zhang Z.M., Tian Y.J., Wang B.J., Yang Y., Zhao X.P., Lu M.J., Gong F.L., Yang D.L. (2006). Inhibition of hepatitis B virus replication by APOBEC3G in vitro and in vivo. World J. Gastroenterol..

[95] Noguchi C., Hiraga N., Mori N., Tsuge M., Imamura M., Takahashi S., Fujimoto Y., Ochi H., Abe H., Maekawa T. (2007). Dual effect of APOBEC3G on Hepatitis B virus. J. Gen. Virol..

[96] Nguyen D.H., Gummuluru S., Hu J. (2007). Deamination-Independent Inhibition of Hepatitis B Virus Reverse Transcription by APOBEC3G. J. Virol..

[97] Hakata Y., Miyazawa M. (2020). Deaminase-Independent Mode of Antiretroviral Action in Human and Mouse APOBEC3 Proteins. Microorganisms.

[98] Bonvin M., Achermann F., Greeve I., Stroka D., Keogh A., Inderbitzin D., Candinas D., Sommer P., Wain-Hobson S., Vartanian J.-P. (2006). Interferon-inducible expression of APOBEC3 editing enzymes in human hepatocytes and inhibition of hepatitis B virus replication. Hepatology.

[99] Lucifora J., Xia Y., Reisinger F., Zhang K., Stadler D., Cheng X., Sprinzl M.F., Koppensteiner H., Makowska Z., Volz T. (2014). Specific and Nonhepatotoxic Degradation of Nuclear Hepatitis B Virus cccDNA. Science.

[100] Xia Y., Stadler D., Lucifora J., Reisinger F., Webb D., Hosel M., Michler T., Wisskirchen K., Cheng X., Zhang K. (2016). Interferon-gamma and Tumor Necrosis Factor-alpha Produced by T Cells Reduce the HBV Persistence Form, cccDNA, without Cytolysis. Gastroenterology.

[101] Jost M., Santos D.A., Saunders R.A., Horlbeck M.A., Hawkins J.S., Scaria S.M., Norman T.M., Hussmann J.A., Liem C.R., Gross C.A. (2020). Titrating gene expression using libraries of systematically attenuated CRISPR guide RNAs. Nat. Biotechnol..

[102] Kostyushev D., Brezgin S., Kostyusheva A., Ponomareva N., Bayurova E., Zakirova N., Kondrashova A., Goptar I., Nikiforova A., Sudina A. (2023). Transient and tunable CRISPRa regulation of APOBEC/AID genes for targeting hepatitis B virus. Mol. Ther.-Nucleic Acids.

[103] Coggins S.A., Mahboubi B., Schinazi R.F., Kim B. (2020). SAMHD1 Functions and Human Diseases. Viruses.

[104] Deutschmann J., Gramberg T. (2021). SAMHD1 … and Viral Ways around It. Viruses.

[105] Chen Z., Zhu M., Pan X., Zhu Y., Yan H., Jiang T., Shen Y., Dong X., Zheng N., Lu J. (2014). Inhibition of Hepatitis B virus replication by SAMHD1. Biochem. Biophys. Res. Commun..

[106] Sommer A.F.R., Rivière L., Qu B., Schott K., Riess M., Ni Y., Shepard C., Schnellbächer E., Finkernagel M., Himmelsbach K. (2016). Restrictive influence of SAMHD1 on Hepatitis B Virus life cycle. Sci. Rep..

[107] Jeong G.U., Park I.H., Ahn K., Ahn B.Y. (2016). Inhibition of hepatitis B virus replication by a dNTPase-dependent function of the host restriction factor SAMHD1. Virology.

[108] Cohen D., Adamovich Y., Reuven N., Shaul Y. (2009). Hepatitis B virus activates deoxynucleotide synthesis in nondividing hepatocytes by targeting the R2 gene. Hepatology.

[109] Ricardo-Lax I., Ramanan V., Michailidis E., Shamia T., Reuven N., Rice C.M., Shlomai A., Shaul Y. (2015). Hepatitis B virus induces RNR-R2 expression via DNA damage response activation. J. Hepatol..

[110] Perez-Caballero D., Zang T., Ebrahimi A., McNatt M.W., Gregory D.A., Johnson M.C., Bieniasz P.D. (2009). Tetherin Inhibits HIV-1 Release by Directly Tethering Virions to Cells. Cell.

[111] Mahauad-Fernandez W.D., Okeoma C.M. (2016). The role of BST-2/Tetherin in host protection and disease manifestation. Immun. Inflamm. Dis..

[112] Yan R., Zhao X., Cai D., Liu Y., Block T.M., Guo J.-T., Guo H. (2015). The Interferon-Inducible Protein Tetherin Inhibits Hepatitis B Virus Virion Secretion. J. Virol..

[113] Lv M., Zhang B., Shi Y., Han Z., Zhang Y., Zhou Y., Zhang W., Niu J., Yu X.F. (2015). Identification of BST-2/tetherin-induced hepatitis B virus restriction and hepatocyte-specific BST-2 inactivation. Sci. Rep..

[114] Miyakawa K., Matsunaga S., Watashi K., Sugiyama M., Kimura H., Yamamoto N., Mizokami M., Wakita T., Ryo A. (2015). Molecular dissection of HBV evasion from restriction factor tetherin: A new perspective for antiviral cell therapy. Oncotarget.

[115] Inuzuka M., Hayakawa M., Ingi T. (2005). Serinc, an Activity-regulated Protein Family, Incorporates Serine into Membrane Lipid Synthesis. J. Biol. Chem..

[116] Liu Y., Wang H., Zhang J., Yang J., Bai L., Zheng B., Zheng T., Wang Y., Li J., Zhang W. (2020). SERINC5 Inhibits the Secretion of Complete and Genome-Free Hepatitis B Virions Through Interfering With the Glycosylation of the HBV Envelope. Front. Microbiol..

[117] Minor M.M., Hollinger F.B., McNees A.L., Jung S.Y., Jain A., Hyser J.M., Bissig K.-D., Slagle B.L. (2020). Hepatitis B Virus HBx Protein Mediates the Degradation of Host Restriction Factors through the Cullin 4 DDB1 E3 Ubiquitin Ligase Complex. Cells.

[118] Lebossé F., Inchauspé A., Locatelli M., Miaglia C., Diederichs A., Fresquet J., Chapus F., Hamed K., Testoni B., Zoulim F. (2020). Quantification and epigenetic evaluation of the residual pool of hepatitis B covalently closed circular DNA in long-term nucleoside analogue-treated patients. Sci. Rep..

[119] Balagopal A., Grudda T., Ribeiro R.M., Saad Y.S., Hwang H.S., Quinn J., Murphy M., Ward K., Sterling R.K., Zhang Y. (2020). Single hepatocytes show persistence and transcriptional inactivity of hepatitis B. J. Clin. Investig..

[120] Balagopal A., Hwang H.S., Grudda T., Quinn J., Sterling R.K., Sulkowski M.S., Thio C.L. (2020). Single Hepatocyte Hepatitis B Virus Transcriptional Landscape in HIV Coinfection. J. Infect. Dis..

[121] Grudda T., Hwang H.S., Taddese M., Quinn J., Sulkowski M.S., Sterling R.K., Balagopal A., Thio C.L. (2022). Inte-grated hepatitis B virus DNA maintains surface antigen production during antiviral treatment. J. Clin. Investig..

[122] Thio C.L., Taddese M., Saad Y., Zambo K., Ribeiro R.M., Grudda T., Sulkowski M.S., Sterling R.K., Zhang Y., Young E.D. (2023). Hepatitis B e Antigen-Negative Single Hepatocyte Analysis Shows Tran-scriptional Silencing and Slow Decay of Infected Cells With Treatment. J. Infect. Dis..

[123] Wooddell C.I., Yuen M.-F., Chan H.L.-Y., Gish R.G., Locarnini S.A., Chavez D., Ferrari C., Given B.D., Hamilton J., Kanner S.B. (2017). RNAi-based treatment of chronically infected patients and chimpanzees reveals that integrated hepatitis B virus DNA is a source of HBsAg. Sci. Transl. Med..

[124] Agarwal K., Buti M., van Bömmel F., Lampertico P., Janczewska E., Bourliere M., Vanwolleghem T., Lenz O., Verbinnen T., Kakuda T.N. (2024). JNJ-73763989 and bersacapavir treatment in nucleos(t)ide analog suppressed patients with chronic hepatitis B: REEF-2. J. Hepatol..

[125] Yuen M.-F., Lim S.-G., Plesniak R., Tsuji K., Janssen H.L., Pojoga C., Gadano A., Popescu C.P., Stepanova T., Asselah T. (2022). Efficacy and Safety of Bepirovirsen in Chronic Hepatitis B Infection. N. Engl. J. Med..

[126] Asselah T., Jacobson I.M., Brunetto M.R., Janssen H.L.A., Takehara T., Hou J.L., Kakuda T.N., Lambrecht T., Beumont M., Kalmeijer R. (2023). Efficacy and safety of the siRNA JNJ-73763989 and the capsid assembly modulator JNJ-56136379 (bersacapavir) with nucleos(t)ide analogues for the treatment of chronic hepatitis B virus infection (REEF-1): A multicentre, double-blind, active-controlled, randomised, phase 2b trial. Lancet Gastroenterol. Hepatol..

